# Terahertz magneto-plasmonics using cobalt subwavelength aperture arrays

**DOI:** 10.1038/s41598-017-12369-5

**Published:** 2017-09-20

**Authors:** Barun Gupta, Shashank Pandey, Anjali Nahata, Berardi Sensale-Rodriguez, Sivaraman Guruswamy, Ajay Nahata

**Affiliations:** 1 Department of Electrical & Computer Engineering, University of Utah, Salt Lake City, UT 84112 USA; 2 Department of Metallurgical Engineering, University of Utah, Salt Lake City, UT 84112 USA

**Keywords:** Nanophotonics and plasmonics, Terahertz optics

## Abstract

We characterize the terahertz (THz) magneto-plasmonic response of a cobalt-based periodic aperture array. The bare cobalt surface allows for low loss propagation of surface plasmon-polaritons, as evidenced by comparing the reflection from aperture arrays coated with Au and with Co. When an external magnetic field is applied in a polar Kerr geometry, we observe a maximum polarization rotation of ~0.6° and an ellipticity of ~0.35° from the Co-based array. These values are larger than expected based on existing models that include only interband transitions in ferromagnetic metals. We discuss possible reasons for the difference between experiment and theory.

## Introduction

Magneto-optics has been studied extensively over the last several decades, driven largely by interest in technological applications and the ability to understand fundamental properties of materials through spectroscopic studies^1,2^. In the case of ferromagnetic metals, theoretical^3–6^ and experimental^5–12^ studies have been performed almost exclusively at optical frequencies, because the response is assumed to derive primarily from interband transitions. If the metal is appropriately structured, there can also be interactions between the magnetic field response and surface plasmon-polaritons (SPPs)^13–17^. In contrast to Au and Ag, which are commonly used for plasmonics applications in this spectral range, conventional ferromagnetic metals tend to exhibit high losses for SPPs^18,19^, resulting in resonances that are broad or exhibit low quality factors. Because of this, layered structures in which a ferromagnetic metal is sandwiched between thin gold films have become popular^19–23^. If the thickness of the top gold layer is less than the skin depth at the operating wavelength, the SPP electric field can penetrate through to the ferromagnetic layer and allow for magnetic field control of the SPP propagation properties, resulting in a dramatically improved magneto-plasmonic response.

In the terahertz (THz) spectral range, the selection of materials that can be used for plasmonics includes a much broader array of metals because they exhibit larger conductivities than at higher frequencies^18^. All conventional metals and a variety of unconventional metals become useful for THz plasmonics applications, because they allow for sufficiently long SPP propagation lengths. As an example, SPPs have to shown to have a 1/e propagation length of ~12 cm at 0.3 THz on stainless steel^24^. Thus, a plasmonic device fabricated from only a conductive ferromagnetic medium (i.e. not a layered structure) can exhibit a response that is similar to that of an identical device fabricated from a highly conducting metal, such as gold. Nevertheless, there has been relatively little work in the area of THz magneto-plasmonics using ferromagnetic metals.

In this work, we describe the THz magneto-plasmonic properties of a subwavelength aperture array fabricated in a metal foil that is subsequently coated with either gold or cobalt. The measurements are performed using a conventional magneto-optic Kerr effect (MOKE) geometry, in which broadband THz radiation is used as the interrogating radiation. Reflection measurements from the two arrays in the absence of an external magnetic field show nearly identical spectra with only a modest (3%) difference in the resonance amplitude. When a magnetic field is applied, we do not observe any polarization change with the gold array. However, with the cobalt array, we observe a maximum polarization rotation of ~0.6° and a maximum ellipticity of ~0.35°. The hysteresis properties are characterized by a saturation field and coercive field that is consistent with values found in the literature.

## Methods

**Figure 1:**
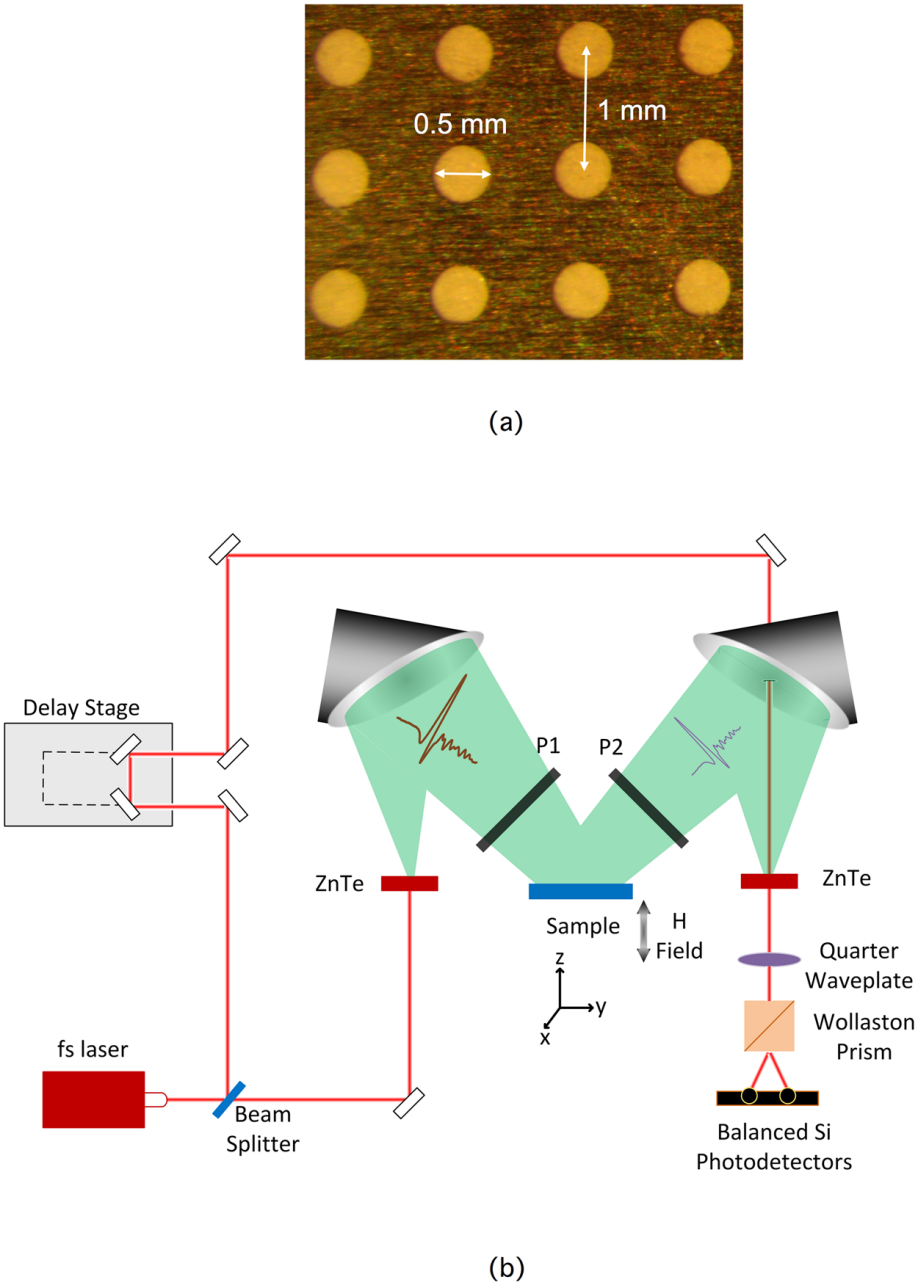
Experimental parameters (**a**) Image of a portion of the aperture array with relevant dimensions. (**b**) Schematic diagram of experimental set up. Broadband s-polarized THz radiation, generated via optical rectification, is incident on the aperture array at 10° with respect to the normal. The magnetic field is applied normal to the sample, which consists of an array of apertures of diameter 0.5 mm with a periodic spacing of 1 mm. Two wire grid polarizers are used to ensure an s-polarized input and measure the s- and p-polarized outputs, independently.

We fabricated periodic arrays of subwavelength apertures in a 5 cm × 5 cm × 75 µm thick stainless steel metal foil via laser ablation using a frequency tripled Nd:YAG laser. Stainless steel was chosen as the substrate medium because it allows for relatively thin free-standing foils that do not easily bend or crease with normal handling. It also allows for relatively inexpensive fabrication of the sample, most notably in comparison to Au. The square lattice consisted of 500 µm diameter apertures with a periodic spacing of 1 mm, as shown in Fig. 1a. For the Au aperture array measurements, we sputter deposited ~1 µm of Au on both sides. We have previously shown that when a structure is overcoated with a metal that is thicker than ~2 skin depths (~300–500 nm total thickness), the SPP properties are determined only by the top metal layer and not by the underlying medium^25^. For the Co aperture array measurements, we sputter deposited ~1 µm of Co over both sides of the entire Au coated array. The sputtered metal also coated the inner surfaces of the apertures with an overall thickness that was greater than 1 µm, because of the double-sided deposition.

The experimental geometry is shown in Fig. 1b. The sample was placed between the poles of an electromagnet, with the magnetic field directed along the z-axis, perpendicular to the metal foil. The magnetic field was calibrated using a Gauss meter in the absence of a sample and was found to vary between ±1600 Gauss. We used conventional THz time-domain spectroscopy^26^ in reflection to measure the magneto-plasmonic properties of the arrays, using nonlinear optical crystals for both generation and coherent detection of broadband THz radiation^27^. Terahertz radiation was incident on the sample at an angle of 10° with respect to the surface normal and vertically polarized (i.e. s-polarized radiation) using a wire grid polarizer. The reflected radiation passed through a second wire grid polarizer that could be rotated to measure independently the s- and p-components of the reflected THz electric field. Reference transmission spectra were taken using a Au-coated unperforated metal foil to allow for direct determination of the absolute amplitude reflection coefficients.

### Data availability

The data that support the findings of this study are available from the corresponding author upon reasonable request.

## Results and Discussion

**Figure 2:**
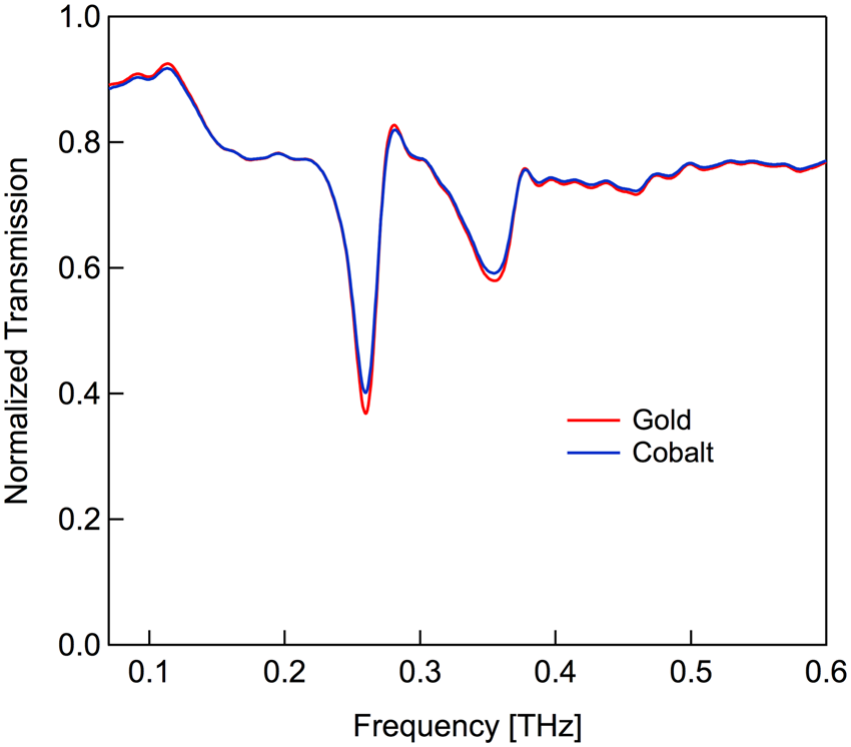
Reflection spectra from the gold and the cobalt aperture arrays in the absence of an applied magnetic field. The measured absolute reflection at the resonance minimum (0.26 THz) is 0.4 for the gold array and 0.37 for the cobalt array.

We first measured the transmission properties of the array for the two different metal coatings in the absence of an applied magnetic field. The amplitude spectra for the two arrays are shown in Fig. 2. The spectra have nearly identical shapes and are characterized by two prominent resonance dips at 0.26 THz and at 0.35 THz. To understand this, we consider the excitation of surface plasmon-polaritons (SPPs) by incident light. The periodicity of the apertures allows for conservation of momentum, which can be written as^28^

1 $${k}_{SPP}={k}_{x}\pm i{G}_{x}\pm i{G}_{y},$$

where $|{{\rm{k}}}_{{\rm{x}}}|=(2{\rm{\pi }}/{\rm{\lambda }})$sinθ is the component of the wave vector of the incident light in the plane of the sample, G_x_ and G_y_ are reciprocal lattice vectors associated with the array (for a square array, G_x_ = G_y_ = 2π/P, where P is the aperture periodicity), and i and j are integers. For transmission measurements of these aperture arrays at normal incidence, the frequency location of dips on the high frequency side of the resonances, ν_ij_, can be modeled using the relation^29^

2 $${\nu }_{ij}=\frac{c\sqrt{{i}^{2}+{j}^{2}}}{P}(\frac{{\varepsilon }_{m}{\varepsilon }_{d}}{{\varepsilon }_{m}+{\varepsilon }_{d}})=\frac{c\sqrt{{i}^{2}+{j}^{2}}}{P}{n}_{SPP},$$

where c is the speed of light in vacuum, ε_m_ is the complex dielectric constant of the metal, and ε_d_ is the dielectric constant of the adjacent dielectric and n_SPP_ is the effective refractive index for SPPs. For the aperture array using either gold or cobalt, n_SPP_ ≅ 1 (see ref.^24^ and Supplementary Information). Thus, with P = 1 mm, we expect transmission minima at 0.3 THz associated with the ( ± 1, 0) resonance and at 0.42 THz associated with the (±1, ±1) resonance. The transmission peaks, which occur at slightly lower frequencies, correspond to dips in the reflection spectra and are in good general agreement with expectations. For a 10° angle of incidence with s-polarized incident radiation, the lowest order resonance shifts slightly to higher frequencies with no additional changes in the spectral shape^30^. A more complete quantitative description of the spectra requires an analysis based on the effective dielectric properties of the array^31^, which is outside the scope of the present work.

It is interesting to note that for the lowest order resonance, there is only a ~3% difference in the absolute reflection between the Au-coated and Co-coated array at 0.26 THz, even though cobalt has a DC conductivity that is approximately a factor of two lower than that of gold. This is consistent with our earlier comment that almost all conventional metals have sufficiently high conductivities in the THz spectral range that the resulting plasmonic response is largely the same^32^. The data also demonstrates that the layered metal approach, often used for magneto-plasmonic studies at optical frequencies, is unnecessary here.

Before moving to the magnetic field induced measurements, we first discuss how the data is presented. The magneto-optic Kerr effect describes the change in the polarization state of light for reflections from a magnetic material. Upon reflection, the incident linearly polarized THz radiation experiences both a rotation of the polarization plane, given by θ_K_, and an ellipticity, given by ε_K_, that corresponds to the phase difference between the parallel and perpendicular components of the electric field in the plane of incidence. These two components can be connected to experimentally measured electric field reflection coefficients through the relationship^2^

3 $${{\rm{\Phi }}}_{k}={\theta }_{k}+i{\varepsilon }_{k}=\frac{{r}_{ps}}{{r}_{ss}},$$

where Φ_K_ is the complex Kerr angle, r_ps_ is the reflection coefficient for an s-polarized incident wave and p-polarized reflected wave, r_ss_ is the reflection coefficient for an s-polarized incident wave and s-polarized reflected wave. In contrast to measurements at optical frequencies, time-domain THz spectroscopy yields both the amplitude and phase of the reflected THz beam, allowing for straightforward determination of both angles. An equivalent relationship can be written if p-polarized incident radiation is used.

**Figure 3:**
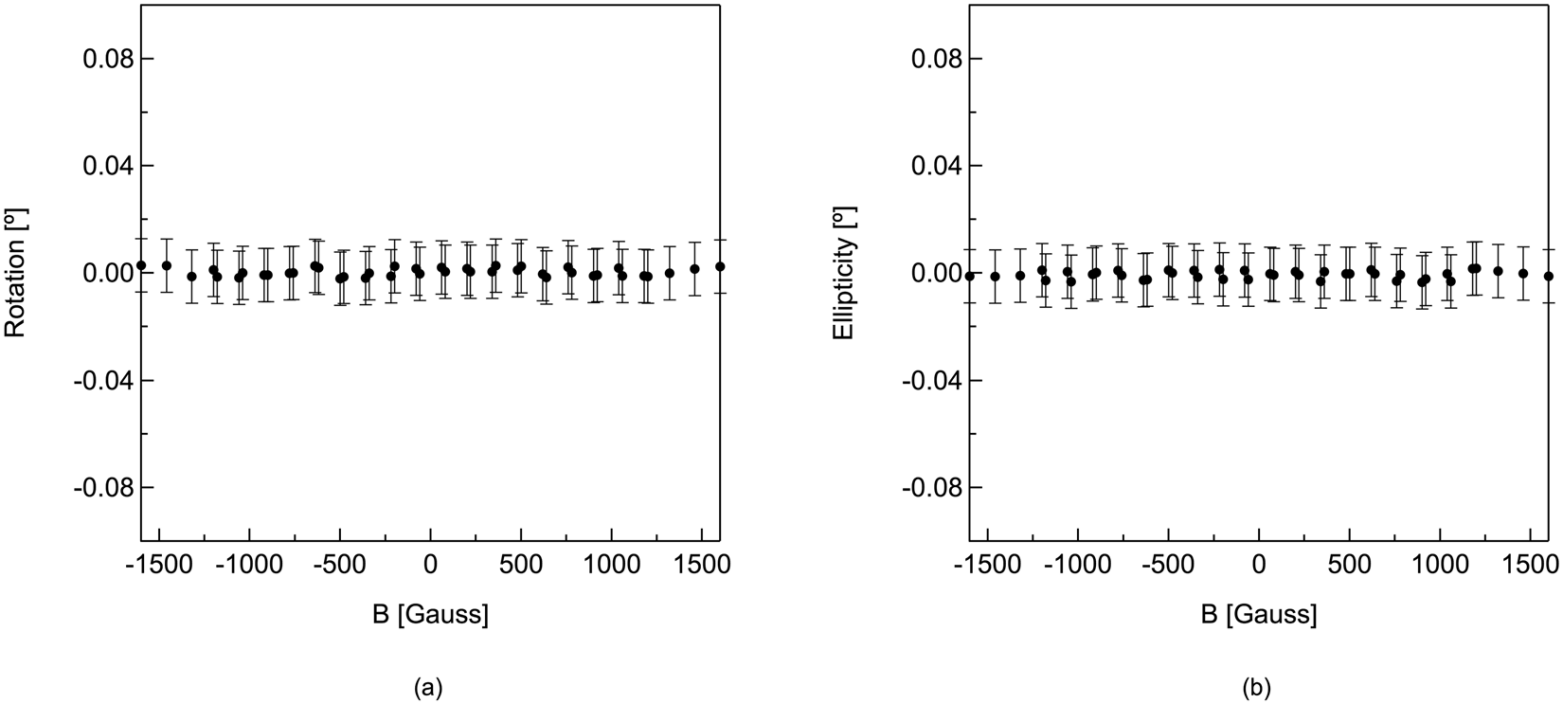
Magnetic field response for the gold-coated array at 0.26 THz. (**a**) Measured polarization rotation of the reflected THz radiation for applied magnetic fields ranging from +1600 Gauss to −1600 Gauss (**b**) Measured ellipticity of the reflected THz radiation for applied magnetic fields ranging from +1600 Gauss to −1600 Gauss.

In Fig. 3, we show the two components of the magnetic field induced complex Kerr angle at 0.26 THz, corresponding to the frequency of the lowest order resonance dip for the Au-coated aperture array. The measured polarization rotation, θ_K_, is shown in Fig. 3(a), and the measured ellipticity, ε_K_, is shown in Fig. 3(b). In both sets of data, there does not appear to be any recognizable pattern in the response as a function of the applied magnetic field. This is generally consistent with earlier measurements using Au and other diamagnetic materials. Thus, the data represents the noise level or a minimum resolvable angle of 0.003° or 5 × 10^−5^ radians from a single measurement. The error bars are determined from 25 independent measurements, demonstrating consistency in the observations.

**Figure 4:**
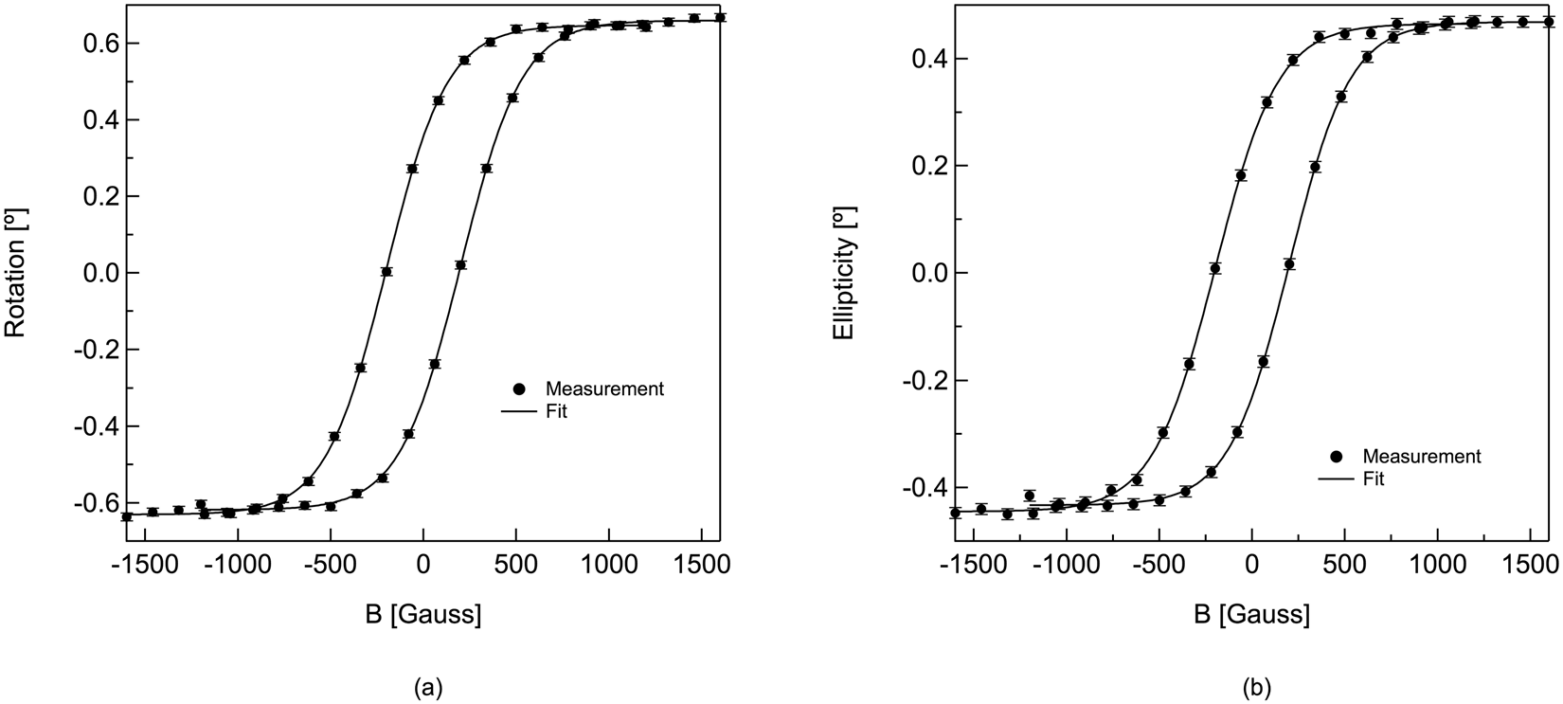
Magnetic field response for the cobalt-coated array at 0.26 THz. (**a**) Measured polarization rotation of the reflected THz radiation for applied magnetic fields ranging from +1600 Gauss to −1600 Gauss (**b**) Measured ellipticity of the reflected THz radiation for applied magnetic fields ranging from +1600 Gauss to −1600 Gauss. The data are fit to a logistic (sigmoid) function of the form f(x) = A + B/(1 + C exp (Dx)), where A, B, C and D are constants. The error bars are approximately the same size as the filled circle markers in both plots.

After coating the array with cobalt, we once again measured complex Kerr angle, with the polarization rotation, θ_K_, shown in Fig. 4(a), and the measured ellipticity, ε_K_, shown in Fig. 4(b). Both angles are seen to be proportional to the magnetization, which is consistent with measurements in a polar Kerr geometry. From these data, the magnetic coercivity is found to be ~300 Gauss and saturation magnetic field is found to be ~1100 Gauss. In the cobalt aperture arrays, for example, the maximum ellipticity at λ = 824 nm was found to be ~0.3°. For comparison, the observed polarization rotation and ellipticity with a planar (unperforated) cobalt coated stainless steel foil was below our detection limit.

Measurements of the Kerr angle in the visible spectral range typically yield values of less than 1°. These values are in reasonable agreement with *ab initio* calculations that only take interband contributions to the optical conductivity into account^3^. Intraband contributions, which are assumed to be well-approximated by an empirical Drude conductivity, normally only contribute to the Kerr angle at low frequencies and are typically neglected in first principles calculations. However, recent measurements have suggested that the Drude model may not accurately model the conductivity for metals in the THz spectral range^24,33^. It is also worth noting that Kerr effect measurements on planar ferromagnetic films and perforated films are unlikely to yield similar results. In the case of an aperture array, the incident radiation is coupled to SPPs that propagate along the surface before a fraction of the radiation is reflected in the specular reflection direction. In contrast to cobalt arrays designed for visible frequencies, where the propagation length is short because of the high dielectric losses, the propagation length along such arrays at THz frequencies can be on the order of several cm, allowing for extended interaction between the THz radiation and the applied magnetic field. Theoretical work is needed to fully account for these issues.

## Conclusion

In conclusion, we have demonstrated that ferromagnetic metals are an attractive class of materials for magneto-plasmonic studies in the THz spectral range. Using a Co-coated aperture arrays, we find that the reflection spectrum is very similar to that of an identical Au-coated array. At 0.26 THz, corresponding to the minimum in the lowest order resonance, there is only a 3% absolute difference in reflection between the two arrays, even though the DC conductivity differs by a factor of two between these two metals, demonstrating that a more complex layered metal geometry is not necessary to probe the magneto-plasmonic response of this medium. When a magnetic field is applied normal to the array surface, the polarization of the reflected THz radiation exhibits both a large rotation and ellipticity. In fact, these values are larger than are expected typically from *ab initio* calculations. However, these calculations assume simple reflection from the metal surface, corresponding to an extremely small interaction length (i.e. the skin depth of the metal). It is possible that the larger observed values arise from interactions between the THz SPPs propagating along the cobalt surface and the applied magnetic fields. Further experimental and theoretical work is needed to understand these results.

## Electronic supplementary material

Supplementary Information
