# Editorial Expression of Concern: Terahertz magneto-plasmonics using cobalt subwavelength aperture arrays

**DOI:** 10.1038/s41598-022-10847-z

**Published:** 2022-04-29

**Authors:** Barun Gupta, Shashank Pandey, Anjali Nahata, Berardi Sensale-Rodriguez, Sivaraman Guruswamy, Ajay Nahata

**Affiliations:** 1 Department of Electrical & Computer Engineering, University of Utah, Salt Lake City, UT 84112 USA; 2 Department of Metallurgical Engineering, University of Utah, Salt Lake City, UT 84112 USA

Editorial Expression of Concern to: *Scientific Reports* 10.1038/s41598-017-12369-5, published online 20 September 2017

The Editors are issuing an Editorial Expression of Concern for this Article.

After publication concerns were raised with respect to the integrity of the magnetic data reported. Editors contacted the Authors to request the raw data, but the corresponding author could not be reached and other Authors informed the journal that they had not been involved in the data collection. Editors subsequently contacted the University of Utah, which confirmed that the raw data for this part of the study are not available. Consequently, readers are advised to interpret the results and conclusions presented in this Article with caution.

Berardi Sensale-Rodriguez agrees to this Editorial Expression of Concern. Barun Gupta, Shashank Pandey, Anjali Nahata, Sivaraman Guruswamy & Ajay Nahata have not responded to correspondence about this Editorial Expression of Concern.

