# Supplementary material for: Terahertz magneto-plasmonics using cobalt subwavelength aperture arrays
Source: Sci Rep. 2017 Sep 20;7:12019. doi: 10.1038/s41598-017-12369-5 (PMC5607252; doi:10.1038/s41598-017-12369-5)
Supplement: Supplementary file 1 — Supplementary Information [file 41598_2017_12369_MOESM1_ESM.pdf]

Supplementary Information

Terahertz magneto-plasmonics using cobalt subwavelength aperture arrays

Barun Gupta,[1] Shashank Pandey,[1] Anjali Nahata,[1] Berardi Sensale-Rodriguez,[1] Sivaraman Guruswamy,[2] and Ajay Nahata[1]

[1] Department of Electrical & Computer Engineering, University of Utah, Salt Lake City, UT 84112 USA

[2] Department of Metallurgical Engineering, University of Utah, Salt Lake City, UT 84112 USA

[*] Correspondence and requests for materials should be sent to A.N. (E-mail: nahata@ece.utah.edu)

Measurement of the THz Dielectric Properties of Cobalt

We coated a 400 μm thick stainless steel foil with ~1 μm of cobalt. This film thickness is sufficiently thick the electric field does not penetrate to the underlying medium [S1]. We used a modified THz time-domain spectroscopy system to characterize the properties of a cobalt coated stainless steel foil. Broadband THz radiation was generated using a 1 mm thick <110> ZnTe crystal and then collected and collimated using an off-axis paraboloidal mirror. The radiation was focused using a 150 mm TPX lens onto a straight 300 μm wide by 100 μm deep rectangular cross-section groove that was 2 cm in length and fabricated in a 400 μm thick stainless steel metal foil. The groove acted as a coupler by scattering a fraction of the incident radiation into SPPs [S2]. This input coupler was physically abutted to the cobalt coated foil. The small separation between the two pieces, coupler and sample, had minimal effect on the propagation

properties of the coupled SPPs. The time-domain properties of the z-component of the propagating THz electric field were measured using a second <110> ZnTe detection crystal via electro-optic sampling [S3]. The temporal resolution available with our apparatus was 6.66 fs. This crystal could be freely moved about the surface of the sample, as well along the z-axis, allowing for the electric field in the half-space above the metal surface to be completely mapped. We measured THz time-domain waveforms at multiple positions along the length of the cobalt-coated foil. Extraction of the dielectric properties of the cobalt film from the time-domain data is described in detail in [S4].

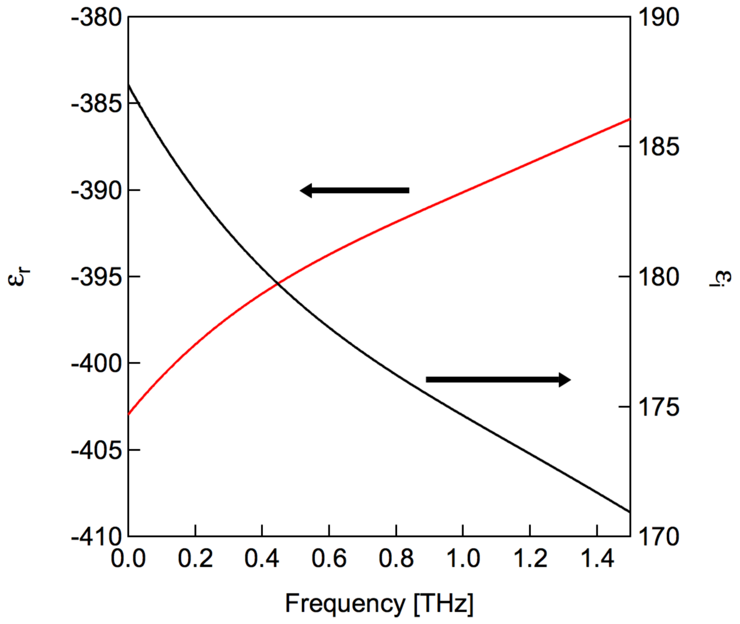

Figure S1. Complex dielectric properties of cobalt

References

[S1] Shou, X., Agrawal, A. & Nahata, A. Role of metal film thickness on the enhanced transmission properties of a periodic array of subwavelength apertures. *Opt. Express* **13,** 9834–9840 (2005).

[S2] Zhu, W., Agrawal, A. & Nahata, A. Planar plasmonic terahertz guided-wave devices. *Opt. Express* **16,** 6216–6226 (2008).

[S3] Nahata, A., Weling, A. S. & Heinz, T. F. A wideband coherent terahertz spectroscopy system using optical rectification and electro-optic sampling. *Appl. Phys. Lett.* **69,** 2321–2323 (1996).

[S4] Pandey, S., Liu, S., Gupta, B. & Nahata, A. Self-referenced measurements of the dielectric properties of metals using terahertz time-domain spectroscopy via the excitation of surface plasmon-polaritons. *Photon. Res.* **1,** 148–153 (2013).
